# Kidney replacement therapy for men and women according to the ERA Registry and the USRDS

**DOI:** 10.1093/ndt/gfaf233

**Published:** 2025-10-31

**Authors:** Vianda S Stel, Nicholas C Chesnaye, Rianne Boenink, Brittany A Boerstra, Megan E Astley, Shona Methven, Line Heylen, Halima Resic, Marc A G J ten Dam, Kristine Hommel, Marit D Solbu, Maria F Slon Roblero, Nuria Aresté-Fosalba, Danilo Radunovic, Héctor García López, Lukas Buchwinkler, Rebecca Guidotti, Mathilde Lassalle, Carmen Santiuste, Maria Stendahl, Olafur S Indridason, Almudena Escribá, María Encarnación Bouzas-Caamaño, Olga Lucía Rodriguez Arévalo, George Moustakas, Hermann Hernández Vargas, Alberto Ortiz, Anneke Kramer

**Affiliations:** ERA Registry, Department of Medical Informatics, Amsterdam UMC - location University of Amsterdam, Amsterdam, The Netherlands; Amsterdam Public Health Research Institute, Quality of Care, Amsterdam, The Netherlands; ERA Registry, Department of Medical Informatics, Amsterdam UMC - location University of Amsterdam, Amsterdam, The Netherlands; Amsterdam Public Health Research Institute, Quality of Care, Amsterdam, The Netherlands; ERA Registry, Department of Medical Informatics, Amsterdam UMC - location University of Amsterdam, Amsterdam, The Netherlands; Amsterdam Public Health Research Institute, Quality of Care, Amsterdam, The Netherlands; ERA Registry, Department of Medical Informatics, Amsterdam UMC - location University of Amsterdam, Amsterdam, The Netherlands; Amsterdam Public Health Research Institute, Quality of Care, Amsterdam, The Netherlands; ERA Registry, Department of Medical Informatics, Amsterdam UMC - location University of Amsterdam, Amsterdam, The Netherlands; Amsterdam Public Health Research Institute, Quality of Care, Amsterdam, The Netherlands; Scottish Renal Registry, Public Health Scotland, Edinburgh, Scotland; Renal Unit, Aberdeen Royal Infirmary, Aberdeen, Scotland; Department of Nephrology, Ziekenhuis Oost-Limburg, Genk, Belgium; Hasselt University, Hasselt, Belgium; Society for Nephrology, Dialysis and Transplantation of Bosnia and Herzegovina, Sarajevo, Bosnia and Herzegovina; Dutch Registry RENINE, Nefrovisie, Utrecht, The Netherlands; Department of Nephrology, Holbaek Hospital, Holbaek, Denmark; Section of Nephrology, University Hospital of North Norway, Tromsø, Norway; Metabolic and Renal Research Group, UiT The Arctic University of Norway, Tromsø, Norway; Hospital Universitario de Navarra, Pamplona, Spain; Nephrology Department, Virgen Macarena Hospital, Seville, Spain; Information System of Andalusian Transplant Coordination (SICATA), Seville, Spain; Clinical Center of Montenegro, Clinic for Nephrology, Podgorica, Montenegro; Transplant Autonomic Coordination Department, Health Service of Castilla y León, Castilla y León, Spain; Austrian Dialysis and Transplantation Registry, Department of Internal Medicine IV (Nephrology and Hypertension), Medical University Innsbruck, Innsbruck, Austria; Institute of Nephrology, Stadtspital Zürich, Zürich, Switzerland; Renal Epidemiology and Information Network Registry, Paris, France; Murcia Renal Registry, Department of Epidemiology, Murcia Regional Health Council, IMIB-Arrixaca, Murcia, Spain; CIBER Epidemiología y Salud Pública, Madrid, Spain; Swedish Renal Registry, Ryhov Regional Hospital, Jonkoping, Sweden; Section of Nephrology, Landspitali University Hospital, Reykjavik, Iceland; Regional Office for Transplant Coordination, Community of Madrid, Madrid, Spain; Regional Transplant Coordination of Galicia, Galician Health Service, Santiago de Compostela, Spain; Registry of Kidney Patients of the Valencian Community, General Directorate of Public Health, Ministry of Health, Valencia, Spain; PhD Program in Health and Wellness Technologies, Polytechnic University of Valencia, Valencia, Spain; Nephrology Department, General Hospital of Athens “G. Gennimatas”, Athens, Greece; Hospital San Pedro, LogroñoLa, Rioja, Spain; Department of Nephrology and Hypertension, IIS-Fundacion Jimenez Diaz UAM, Madrid, Spain; Department of Medicine, Universidad Autonoma de Madrid, Madrid, Spain; ERA Registry, Department of Medical Informatics, Amsterdam UMC - location University of Amsterdam, Amsterdam, The Netherlands; Amsterdam Public Health Research Institute, Quality of Care, Amsterdam, The Netherlands

**Keywords:** epidemiology, incidence, kidney replacement therapy, mortality, prevalence

## Abstract

**Background:**

This article compares the incidence and prevalence of kidney replacement therapy (KRT), kidney transplantation rates and mortality on KRT between Europe and the USA, including sex comparisons.

**Methods:**

Data were derived for 2022 from the population-based European Renal Association (ERA) Registry and the United States Renal Data System (USRDS).

**Results:**

In 2022, the KRT incidence in the USA [388.7 per million population (pmp)] was 2.7-fold higher than in Europe (146.2 pmp), with a greater difference for women (3.2-fold) than for men (2.4-fold). The proportion of women initiating KRT was lower in Europe (35%) than in the USA (41%). Between 2013 and 2022, the KRT incidence in Europe was stable in women (+0.1% annually) but increased in men (+1.1%). In the USA, the KRT incidence increased similarly in women (+0.2%) and men (+0.3%). On 31 December 2022, the KRT prevalence was 2-fold (women 2.2-fold, men 1.9-fold) higher in the USA (2444.2 pmp) than in Europe (1218.6 pmp). The proportion of women was lower in Europe (38%) than in the USA (41%). The kidney transplantation rate was 1.7-fold higher in the USA (79.1 pmp) than in Europe (45.4 pmp), 1.9-fold for women and 1.7-fold for men, with women accounting for 37% of the recipients versus 39% in the USA. The KRT mortality rate was 1.5 times higher in the USA [145.0 per 1000 patient-years (py)] compared with Europe (100.5 per 1000 py): 1.6-fold for women and 1.4-fold for men. In Europe, mortality was lower for women receiving KRT (93.7 per 1000 py) than for men (104.6 per 1000 py), whereas in the USA the reverse was true (women 148.9 per 1000 py, men 142.2 per 1000 py).

**Conclusion:**

The US had a notably higher KRT incidence, prevalence, kidney transplantation rate and mortality compared with Europe. Differences between Europe and the USA were larger for women than for men.

## Graphical Abstract

**Graphical Abstract:**
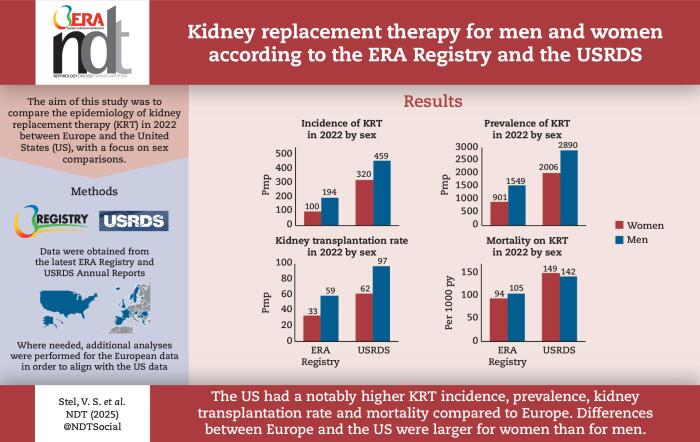

KEY LEARNING POINTS **What was known:**

- Annual reports from the European Renal Association (ERA) Registry and the United States Renal Data System (USRDS) show large international differences in the epidemiology of kidney replacement therapy (KRT).
- The ERA Registry annual reports mainly focus on differences within Europe, while the USRDS annual reports compare data from the USA with other countries.

**This study adds:**

- This study compares the epidemiology of KRT between Europe and the USA.
- In 2022, the USA had a >2.5-fold higher KRT incidence, a 2-fold higher KRT prevalence, an almost 2-fold higher kidney transplantation rate and a 1.5-fold higher KRT mortality compared with Europe.
- In both Europe and the USA, the majority of KRT patients were men. Differences between Europe and the USA were greater for women than for men.

**Potential impact:**

- Renal registries are valuable in identifying disparities in KRT treatment and outcomes across regions and may therefore play an important role in informing policy aimed at reducing inequalities in kidney care.

## INTRODUCTION

Both the European Renal Association (ERA) Registry and the United States Renal Data System (USRDS) collect data on patients with kidney failure who are treated with kidney replacement therapy (KRT), including dialysis and kidney transplantation [1, 2]. Annually, these registries report on the incidence and prevalence of KRT, kidney transplantation rates and patient and graft survival. As these population-based registries capture all patients starting KRT in their regions, they facilitate comparisons of KRT epidemiology across Europe and the USA. While our previous article focused on comparing KRT modalities [3], our present study explores sex-based differences between Europe and the USA. Specifically, our aim is to compare the incidence and prevalence of KRT, kidney transplantation rates and mortality on KRT between Europe and the USA, with a focus on sex comparisons.

## MATERIALS AND METHODS

### Study population and data collection

This article is based on data on the epidemiology of KRT derived from the annual report of the ERA Registry [1] and from reference tables in sections D, E, H and M from the USRDS annual report [2].

The European data include information from 35 national and regional renal registries providing the ERA Registry with individual patient data [1] from the following 17 countries: Austria, Belgium, Bosnia and Herzegovina, Denmark, Estonia, France, Greece, Iceland, Montenegro, The Netherlands, Norway, Romania, Serbia, Spain, Sweden, Switzerland and the UK. Data on paediatric patients were unavailable for Belgium, Montenegro and the Spanish regions of Cantabria, Castile and Léon, Castile-La Mancha and Navarre. Participating renal registries provided data on age at KRT initiation, sex, primary renal disease (PRD), changes in KRT modality and date and cause of death. Informed consent was obtained by each registry in accordance with national and/or regional regulations. Compliance with ethical standards was confirmed by the medical ethical committee of the Amsterdam Medical Centre (W21_123, No. 21.136). Population data were obtained from the statistical office of the European Union (Eurostat) [4] or via national statistics agencies. More information on the study population and data collection can be found in the ERA Registry and USRDS annual reports [1, 2].

### Analyses

KRT incidence was defined as the number of patients starting dialysis or receiving a pre-emptive kidney transplant in a given year, calculated per million population (pmp) using the mid-year general population as the denominator. Most patients begin with dialysis, while usually only a small proportion receive a pre-emptive kidney transplant, meaning they undergo transplantation without prior dialysis. KRT prevalence was defined as the number of patients receiving dialysis or kidney transplantation on 31 December in a given year and the kidney transplantation rate was defined as the number of patients receiving a pre-emptive or non-pre-emptive kidney transplant in a year, both calculated per million population. To ensure comparability with the US data, the age categories used for the ERA Registry data were aligned with those in the US data.

Mortality rates were calculated for each year as the number of patients who died that year while receiving KRT, divided by the total time-at-risk-of-death for all patients on KRT in the same year, and presented per 1000 patient-years (py). The total time-at-risk-of-death on KRT for an individual was the time between 1 January (for prevalent patients in the previous year) or the date of KRT initiation (for incident patients) until the date of death, date of loss to follow-up, 90 days after recovery of renal function or 31 December.

## RESULTS

### Incidence of KRT

**Figure 1:**
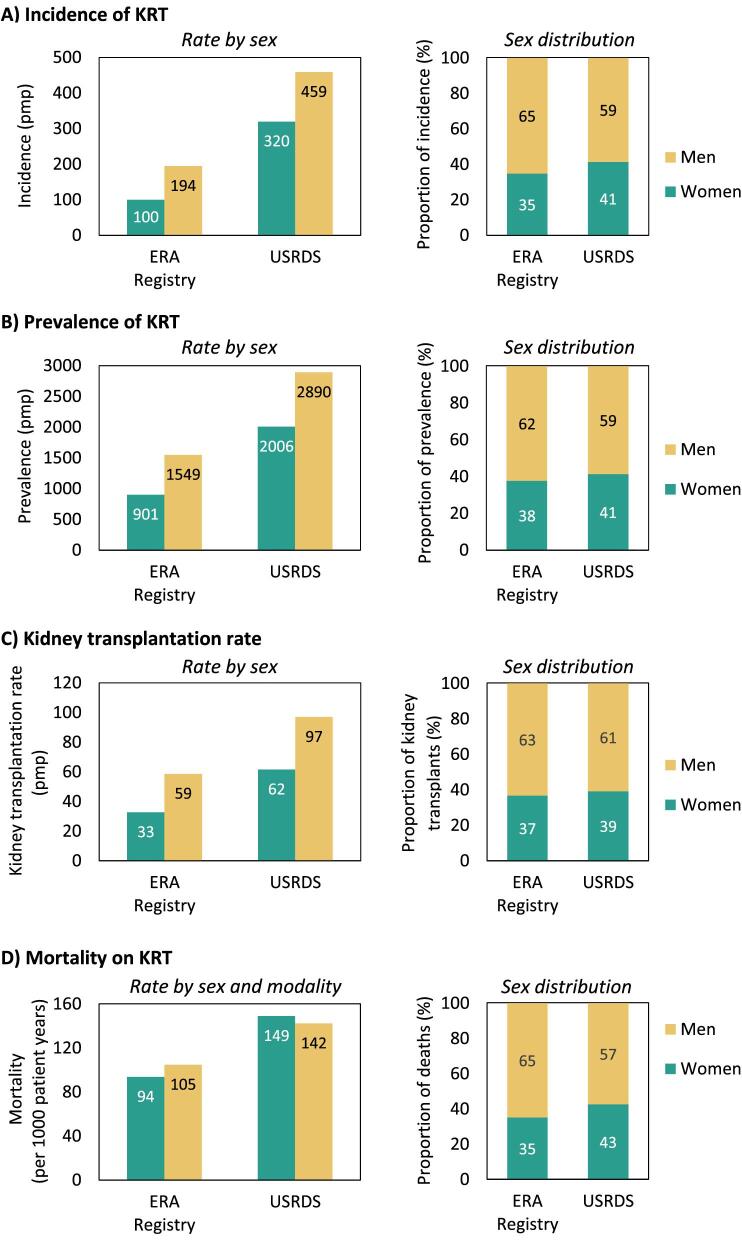
The **(A)** incidence of KRT, **(B)** prevalence of KRT on 31 December, **(C)** kidney transplantation and **(D)** mortality on KRT in 2022, as rate per million population or per 1000 py and as a proportion of all patients, by registry.

In 2022, 41 813 patients in Europe (population 286 million) and 131 194 patients in the USA (population 335 million) initiated KRT, corresponding to a KRT incidence of 146.2 pmp and 388.7 pmp, respectively (Table 1). This reflects a >2.5-fold higher KRT incidence in the USA compared with Europe. The difference was greater for women than for men (3.2-fold versus 2.4-fold). In both Europe and the USA, fewer women initiated KRT (Europe 99.9 pmp, USA 319.8 pmp) than men (Europe 194.4 pmp, USA 458.8 pmp), however, the percentage of women initiating KRT was lower in Europe (35%) than in the USA (41%) (Fig. 1A).

**Table 1:** Trends in the incidence and prevalence of KRT, kidney transplantation rates and mortality on KRT from the ERA Registry and the USRDS.

		Yearly rate										
Data	Type	2013	2014	2015	2016	2017	2018	2019	2020	2021	2022	Average annual percent change, 2013–2022
**Incidence of KRT (pmp)**												
ERA Registry	All	137.4	141.4	143.1	145.8	147.6	147.4	149.2	139.8	148.1	146.2	+0.7
	Women	99.3	100.5	101.7	102.5	103.2	103.3	104.3	95.7	101.1	99.9	+0.1
	Men	177.1	183.7	186.1	190.8	193.6	193.2	195.9	185.5	197.0	194.4	+1.1
USRDS	All	380.5	387.8	400.1	400.2	396.7	402.7	412.3	397.2	407.4	388.7	+0.3
	Women	316.7	321.5	331.6	333.5	327.7	330.6	338.4	325.2	338.5	319.8	+0.2
	Men	446.4	456.2	470.8	468.9	467.8	477.0	488.4	471.5	477.3	458.8	+0.3
**Prevalence of KRT (pmp)**												
ERA Registry	All	1019.1	1051.8	1077.8	1103.4	1133.8	1156.6	1177.6	1178.8	1190.9	1218.6	+2.0
	Women	777.1	797.8	813.3	829.7	849.7	865.4	879.4	878.2	882.6	901.1	+1.7
	Men	1270.9	1315.8	1352.4	1387.5	1428.7	1458.8	1487.0	1490.3	1511.7	1548.6	+2.2
USRDS	All	2091.3	2155.8	2221.4	2281.7	2336.3	2397.3	2465.4	2446.4	2438.1	2444.2	+1.8
	Women	1759.1	1804.8	1853.0	1897.6	1934.3	1976.5	2026.1	2013.5	2010.2	2006.0	+1.5
	Men	2434.2	2517.8	2601.3	2677.6	2750.7	2831.1	2918.3	2889.4	2872.9	2889.8	+1.9
**Kidney transplantation rate (pmp)**												
ERA Registry	All	43.6	44.3	45.2	46.5	48.6	47.9	48.0	37.1	41.5	45.4	+0.9
	Women	31.3	32.0	33.0	33.5	34.6	34.2	34.7	27.0	29.3	32.7	+0.9
	Men	56.4	57.1	57.9	59.9	63.2	62.1	61.8	47.5	54.2	58.6	+1.0
USRDS	All	56.0	56.0	58.1	61.6	63.6	67.4	74.0	71.8	76.9	79.1	+4.0
	Women	42.7	42.8	44.8	48.4	49.1	52.9	57.2	55.0	59.6	61.5	+4.2
	Men	69.8	69.8	71.9	75.3	78.5	82.4	91.3	89.2	94.4	96.9	+3.8
**Mortality on KRT (per 1000 py)**												
ERA Registry	All	97.5	97.8	99.9	94.1	91.4	96.4	96.7	108.4	105.5	100.5	+0.5
	Women	92.5	94.0	95.5	89.5	84.6	89.7	90.9	101.5	98.8	93.7	+0.3
	Men	100.6	100.2	102.6	97.1	95.7	100.5	100.2	112.6	109.6	104.6	+0.6
USRDS	All	136.2	135.3	136.4	135.2	136.0	134.6	132.2	156.3	157.5	145.0	+0.9
	Women	139.8	138.3	139.2	138.8	139.7	137.6	134.9	157.7	160.1	148.9	+0.9
	Men	133.6	133.0	134.4	132.5	133.3	132.4	130.3	155.2	155.7	142.2	+0.9

**Figure 2:**
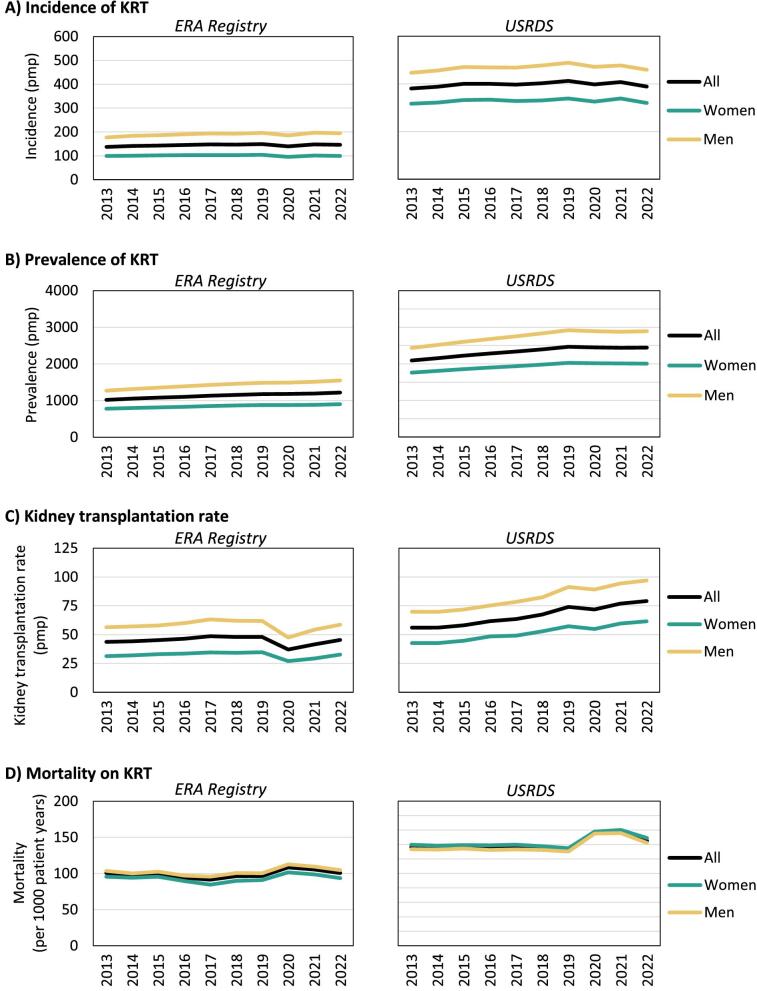
Trends over time in the **(A)** incidence of KRT, **(B)** prevalence of KRT on 31 December, **(C)** kidney transplantation rate per million population and **(D)** mortality on KRT per 1000 py for the ERA Registry and the USRDS, overall and by sex.

Between 2013 and 2022, the overall KRT incidence increased annually by 0.7% in Europe and 0.3% in the USA (Table 1, Fig. 2A). Interestingly, in the USA, the temporal trend was similar for women (+0.2% annually) and men (+0.3%), whereas in Europe the KRT incidence remained almost stable in women (+0.1%) but increased in men (+1.1%).

**Figure 3:**
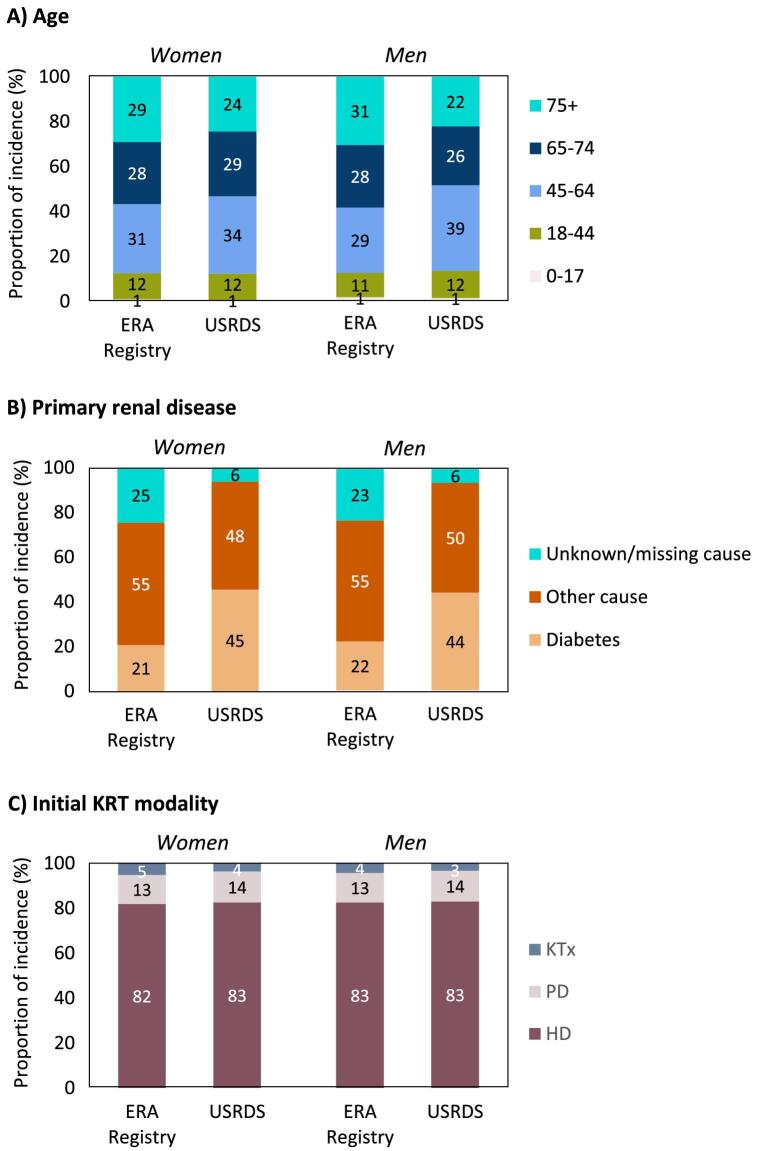
Distribution of **(A)** age, **(B)** diabetes as the primary renal disease and **(C)** initial KRT modality among incident patients on KRT in 2022, by sex and registry. HD: haemodialysis; PD: peritoneal dialysis; KTx: kidney transplantation. Percentages may not add up to 100 due to rounding of numbers.

In 2022, the proportion of older incident KRT patients (≥65 years at the start of KRT) was higher in Europe (58%) compared with the USA (51%). Stratified by sex, this difference in the proportion of incident KRT patients >65 years of age was more pronounced in men (Europe 59%, USA 48%) than in women (Europe 57%, USA 53%). This is the result of a slightly higher proportion of incident KRT patients >65 years of age in men (59%) than in women (57%) in Europe, whereas in the USA this proportion was lower in men (48%) than in women (53%; Fig. 3A).

Although the proportion of patients with diabetes in Europe was about half of that in the USA (22% versus 45%), in both Europe and the USA the proportion of patients with diabetes as the primary renal disease was similar among women and men (Fig. 3B). The distribution of initial KRT modality was similar across both sexes and continents (Fig. 3C).

### Prevalence of KRT

On 31 December 2022, 348 601 patients in participating European countries and 815 896 patients in the USA were receiving KRT, corresponding to KRT prevalences of 1218.6 pmp and 2444.2 pmp, respectively (Table 1). This reflects a 2-fold higher KRT prevalence in the USA compared with Europe. This 2-fold difference was consistent across both sexes, with prevalence rates in Europe of 901.1 pmp for women and 1548.6 pmp for men, compared with 2006.0 pmp and 2889.8 pmp in the USA, respectively, corresponding to fold differences of 2.2 for women and 1.9 for men. The proportion of women receiving KRT was slightly lower in Europe (38%) than in the USA (41%; Fig. 1B).

The annual increase in the KRT prevalence between 2013 and 2022 was similar in Europe (2.0%) and the USA (1.8%), although in the USA the prevalence showed a stabilisation between 2000 and 2022. Stratified by sex, the annual increase was lower for women in both regions (Europe +1.7%, USA +1.5%; Table 1, Fig. 2B) than for men (Europe +2.2%, USA +1.9%).

**Figure 4:**
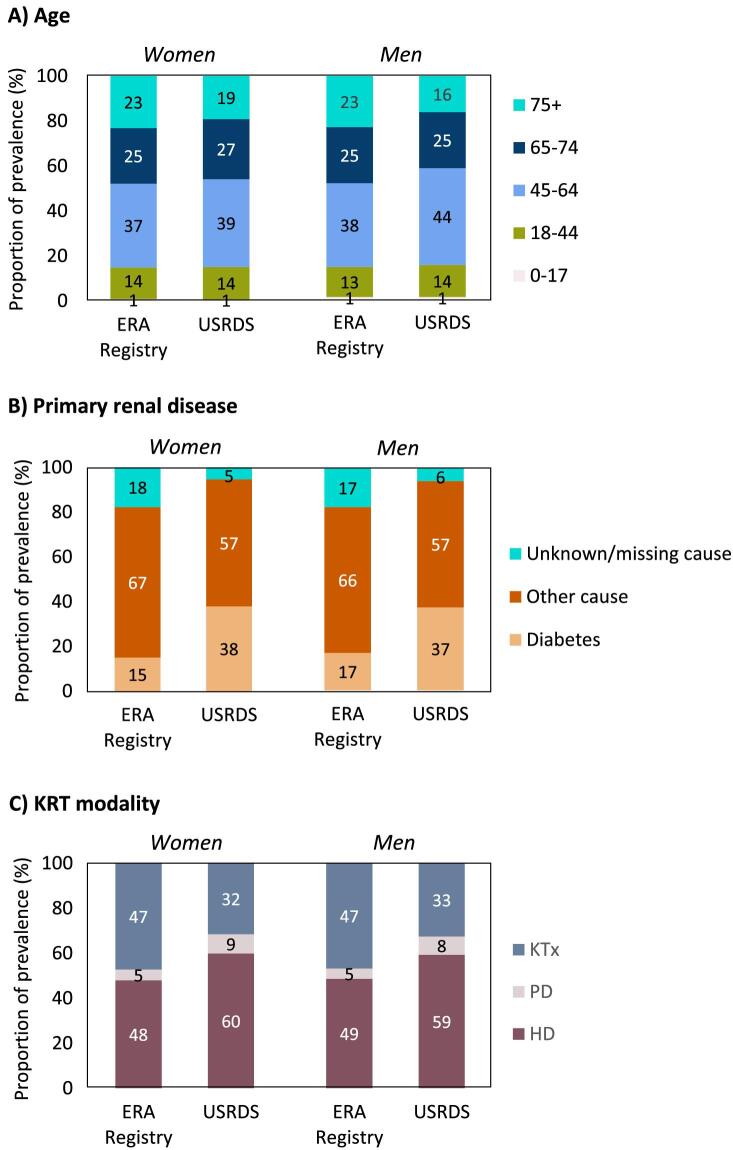
Distribution of **(A)** age, **(B)** diabetes as the primary renal disease and **(C)** KRT modality among prevalent patients receiving KRT on 31 December 2022, by sex and registry. HD: haemodialysis; PD: peritoneal dialysis; KTx: kidney transplantation. Percentages may not add up to 100 due to rounding of numbers.

In 2022, approximately half of both prevalent women and men in Europe were >65 years of age (women 48%, men 48%), whereas in the USA the proportion of patients >65 years of age was higher among women (46%) than men (41%; Fig. 4A). For both sexes, the proportion of prevalent KRT patients with diabetes as primary renal disease was lower in Europe (≈16%) than in the USA (≈37%; Fig. 4B). Conversely, the proportion of prevalent KRT patients living with a functioning transplant was higher in Europe (≈47%) than in the USA (≈33%; Fig. 4C) for both sexes.

### Kidney transplantation

In 2022, 13 042 patients in participating European countries received a kidney transplant (45.4 pmp) compared with 26 362 patients in the USA (79.1 pmp; Table 1). This reflects an almost 2-fold higher kidney transplantation rate in the USA compared with Europe: 1.9-fold for women and 1.7-fold for men. In both Europe and the USA, fewer women (Europe 32.7 pmp, USA 61.5 pmp) received a kidney transplant than men (Europe 58.6 pmp, USA 96.9 pmp), however, the proportion women was slightly lower in Europe (37%) than in the USA (39%; Fig. 1C). Overall, in 2022, 33 kidney transplants were performed per 100 incident women on KRT in Europe and 30 per 100 men. Corresponding figures for the USA were 19 and 21 per 100 incident women and men, respectively.

Between 2013 and 2022, the annual increase in kidney transplantation rates was lower in Europe (+0.9%) than in the USA (+4.0%; Table 1, Fig. 2C). In both Europe and the USA, the coronavirus disease 2019 (COVID-19) pandemic was associated with lower kidney transplantation rates in 2020. In both Europe and the USA, this temporal trend was similar for women (Europe +0.9%, USA +4.2%) and men (Europe +1.0%, USA +3.8%).

### Mortality on KRT

In 2022, 36 226 patients in Europe and 122 797 patients in the USA died while receiving KRT, corresponding to mortality rates of 100.5 and 145.0 per 1000 py at risk (Table 1). This reflects a 1.5-fold higher KRT mortality in the USA compared with Europe: 1.6-fold for women and 1.4-fold for men. While in Europe, mortality was lower for women (93.7 per 1000 py) than for men receiving KRT (104.6 per 1000 py), in the USA the opposite was true (women 148.9 per 1000 py, men: 142.2 per 1000 py) (Fig. 1D). Consequently, KRT mortality in Europe was 37% lower for women and 26% lower for men when compared with the USA.

In both Europe and the USA, KRT mortality rates increased slightly between 2013 and 2019 (+1.3% annually) and increased dramatically in 2020 (+20.5% in Europe: +24.2% in women and +18.5% in men; +32.9% in the USA: +26.3% in women and +37.3% in men), which was primarily due to the COVID-19 pandemic. Mortality rates continued to increase in 2021 in the USA, both in women and in men, while decreasing in 2022, whereas rates decreased for Europe between 2021 and 2022 (Table 1, Fig. 2D).

## DISCUSSION

This study examines the epidemiology of KRT between Europe and the USA, primarily focussing on sex-based comparisons. The incidence of KRT in the USA was >2.5-fold higher than in Europe. At the start of KRT, Europe had a lower proportion of women (35%) compared with the USA (41%). Both the overall KRT prevalence and kidney transplant rate were 2-fold higher in the USA than in Europe, but despite these differences, the female:male ratio was generally similar in both regions, with women representing ≈40% of the population. The KRT mortality rate was 1.5-fold higher in the USA than in Europe. In Europe, men had a higher mortality than women (105 per 1000 py versus 94 per 1000 py), whereas in the USA women had a slightly higher mortality than men (149 per 1000 py versus 142 per 1000 py).

### Incidence of KRT

The potential reasons for the higher incidence of KRT in the USA compared with Europe are discussed in detail in our previously published paper [3]. Regarding sex comparisons, in both Europe and the USA the majority of patients starting KRT were men. Several factors may explain this finding. First, kidney function in men tends to decline faster than in women, increasing their risk of kidney failure [5, 6]. This could be attributed to men generally leading unhealthier lifestyles, and there is some evidence supporting the potentially renoprotective effects of oestrogen in women and/or the damaging effects of testosterone in men [7–10]. Second, people are more likely to die than to reach end-stage kidney failure and start dialysis, and this influences KRT incidence, as mortality acts as a competing risk for KRT. While this does not explain the higher incidence of KRT in men in Europe—where men have a greater mortality risk than women—it could partly account for the higher KRT incidence observed in men in the USA, where women experience higher mortality rates. Third, there may be potential sex differences in treatment choices. For instance, previous studies have shown that older women with kidney failure are two to three times more likely than elderly men to receive conservative care instead of KRT [10–12]. Last, factors relating to access to care and quality of care may also play a role. This includes differences in CKD awareness, as women are reportedly less aware of their CKD than men in the general population [13, 14]. Moreover, a 2022 study from Stockholm demonstrated that among patients with an estimated glomerular filtration rate of <60 ml/min/1.73 m^2^, women were less likely to receive a CKD diagnosis, less likely to have received renin–angiotensin system inhibitors and statins (despite guideline-recommended indications), visited a nephrologist or undergone monitoring of creatinine or albuminuria. These differences persisted irrespective of disease severity, presence of albuminuria or criteria for referral [15]. It is important to note that globally, population-based studies find that the prevalence of CKD stages 3–5 is typically higher in women than in men [8]. The higher prevalence of CKD stage 3–5 in women may be partly attributed to their longer life expectancy combined with the natural decline in kidney function with ageing.

Regarding trends over time, the KRT incidence rate remained stable for women in both Europe and the USA between 2013 and 2022. For men, the incidence rate of KRT also remained stable in the USA during this period but showed a slight increase in Europe. This increase was primarily due to an increasing acceptance of older men on KRT over time [16, 17].

### Prevalence of KRT

In 2022, the prevalence of KRT was twice as high in the USA as it was in Europe with a similar female:male ratio in both regions, with ≈40% women. In Europe, the prevalence of KRT steadily increased for both women and men, whereas in the USA the prevalence stabilized for both sexes since the onset of the COVID-19 pandemic in 2019. An increase in prevalence in Europe indicates that the number of new patients starting KRT in a given year exceeds the number deaths among KRT patients in that same year. In addition to the slight increase in incident men during this period, survival rates have also improved during the past decades (pre-pandemic), mainly in dialysis patients [18]. Despite the somewhat higher mortality rates during and after the pandemic in Europe, prevalence continues to increase, as the number of patients starting KRT exceeds the number of deaths in a given year, which could be explained in part by the influx of incident (older) male patients over time. Likewise, the stabilization of prevalence in the USA since the pandemic began in 2019 means that the number of deaths of patients on KRT seems to be similar to the number of patients starting KRT.

### Kidney transplantation

In 2022, the overall kidney transplantation rate was almost twice as high in the USA as in Europe (79.1 pmp versus 45.4 pmp). In our previously published paper [3] we provide a detailed discussion on why the proportion of prevalent KRT patients living with a functioning graft was lower in the USA despite the higher kidney transplantation rate in the USA compared with Europe.

Regarding sex comparisons, kidney transplantation rates were almost 2-fold higher in men than in women in both Europe and the USA. This difference is likely a consequence of the higher incidence of kidney failure in men compared with women, given that the proportion of prevalent KRT patients living with a kidney transplant is highly similar between sexes, a consistency observed globally [8]. Our study lacks data on the kidney transplant waiting list, meaning that we are unable to determine any sex disparities regarding the eligibility of dialysis patients to receive a kidney transplantation. Although a recently published study from the UK showed no sex differences in waitlisting rates [19], in many countries, including the USA, fewer women, especially older women, were placed on the kidney transplant waiting list than men [8, 20]. Women tend to have better survival rates than men after transplantation, which could explain the similar prevalence of transplantation between both sexes despite fewer women being placed on the waitlist [8].

### Mortality on KRT

In 2022, the mortality on KRT was 1.5-fold higher in the USA than in Europe. Our previously published article also showed that the mortality rate for kidney transplant recipients was approximately one-third higher in the USA compared with Europe, with this gap widening during and after the COVID-19 pandemic. Possible explanations for these regional differences in mortality on KRT and after kidney transplantation are described in that same article [3].

In the general population, women have a survival advantage over men. However, this survival advantage diminishes with declining kidney function [9]. For patients on KRT, this results in very similar survival for both sexes. Specifically, the survival advantage for women on dialysis is reduced to only a few months, and for transplant recipients, it is <1 year [10]. Our results show that KRT mortality was somewhat higher for men in Europe, whereas in the USA the KRT mortality rate was slightly higher for women. Although this discrepancy may be negligible, in Europe this may be partly explained by the higher burden of comorbidities in men starting KRT [21].

### Strengths and limitations

The study’s main strength is that it includes data from almost all dialysis and kidney transplant patients across a large number of European countries (including most Western and some Eastern countries) and the USA. However, due to the wide geographical variation in the epidemiology of KRT, our results may not be generalizable to non-participating European countries or to specific European countries and US states. Moreover, we only present unadjusted results in this article. While both the ERA Registry and USRDS annual reports offer age- and sex-standardized incidence, prevalence and mortality rates, their use of different reference populations makes comparisons difficult. Lastly, while the epidemiology of KRT may have been influenced by the pandemic, this topic is mainly discussed elsewhere [22–25].

## CONCLUSION

In 2022, the incidence of KRT in the USA was >2.5-fold higher than in Europe. Looking at sex comparisons, 41% of new KRT patients in the USA were women compared with 35% in Europe. Despite the USA having higher overall KRT prevalence and kidney transplant rates than Europe, the female:male ratio was generally similar in both regions, with women representing ≈40% of the population. While the KRT mortality rate was 1.5-fold higher in the USA than in Europe, mortality rates on KRT differed between the sexes in both regions. Overall, the differences between Europe and the USA in KRT incidence, prevalence, kidney transplantation rates and mortality were larger for women than for men. These findings represent an important step toward addressing potential sex inequalities. Future research should aim to further explore these disparities in both Europe and the USA with the goal of improving kidney care for all patients.

## Data Availability

Most of the data underlying this article have been published in the ERA Registry Annual Report 2022 and in the USRDS 2024 Annual Report. The remaining data cannot be shared with any third party because the national and regional registries that provided data to the ERA Registry remain the owners of the data.
